# Advances in the Study of the Mechanisms of Physiological Root Resorption in Deciduous Teeth

**DOI:** 10.3389/fped.2022.850826

**Published:** 2022-03-30

**Authors:** Manxue Xiao, Hong Qian, Jingwen Lv, Peixuan Wang

**Affiliations:** Department of Pediatric Dentistry, Stomatological Hospital, Southern Medical University, Guangzhou, China

**Keywords:** deciduous teeth, physiological root resorption, inflammatory microenvironment, mechanical stress, apoptosis

## Abstract

Physiological root resorption of deciduous teeth is a complex physiological process that is essential for the normal replacement of deciduous teeth and permanent teeth in clinical practice, but its importance is often overlooked due to the presence of permanent teeth. This physiological process includes not only the resorption of hard tissues of deciduous teeth, such as dentin and cementum, but also the elimination of soft tissues, such as pulp and periodontal ligament (PDL). However, the mechanisms of physiological root resorption are not yet clear. In this article, the advances of research on the mechanisms related to physiological root resorption will be reviewed in two main aspects: hard tissues and soft tissues of deciduous teeth, specifically in relation to the effects of inflammatory microenvironment and mechanical stress on the resorption of hard tissues, the repair of hard tissues, and the elimination and the histological events of soft tissues.

## Introduction

Under normal circumstances, physiological root resorption and exfoliation of deciduous teeth occur in a certain time and sequence to provide channel and space for the eruption of the permanent successors. In clinical practice, abnormal physiological root resorption is often seen to lead to premature loss or retention of deciduous teeth, which will affect the normal development of the permanent dentition and may cause some problems, such as speech disorders, aesthetic problems and malocclusion (1–3). Therefore, it is of great clinical significance to study the physiological root resorption, which can provide new ideas and theoretical basis for preventing the retention and premature loss of deciduous teeth, or even retaining the deciduous teeth without successors.

**Figure 1:**
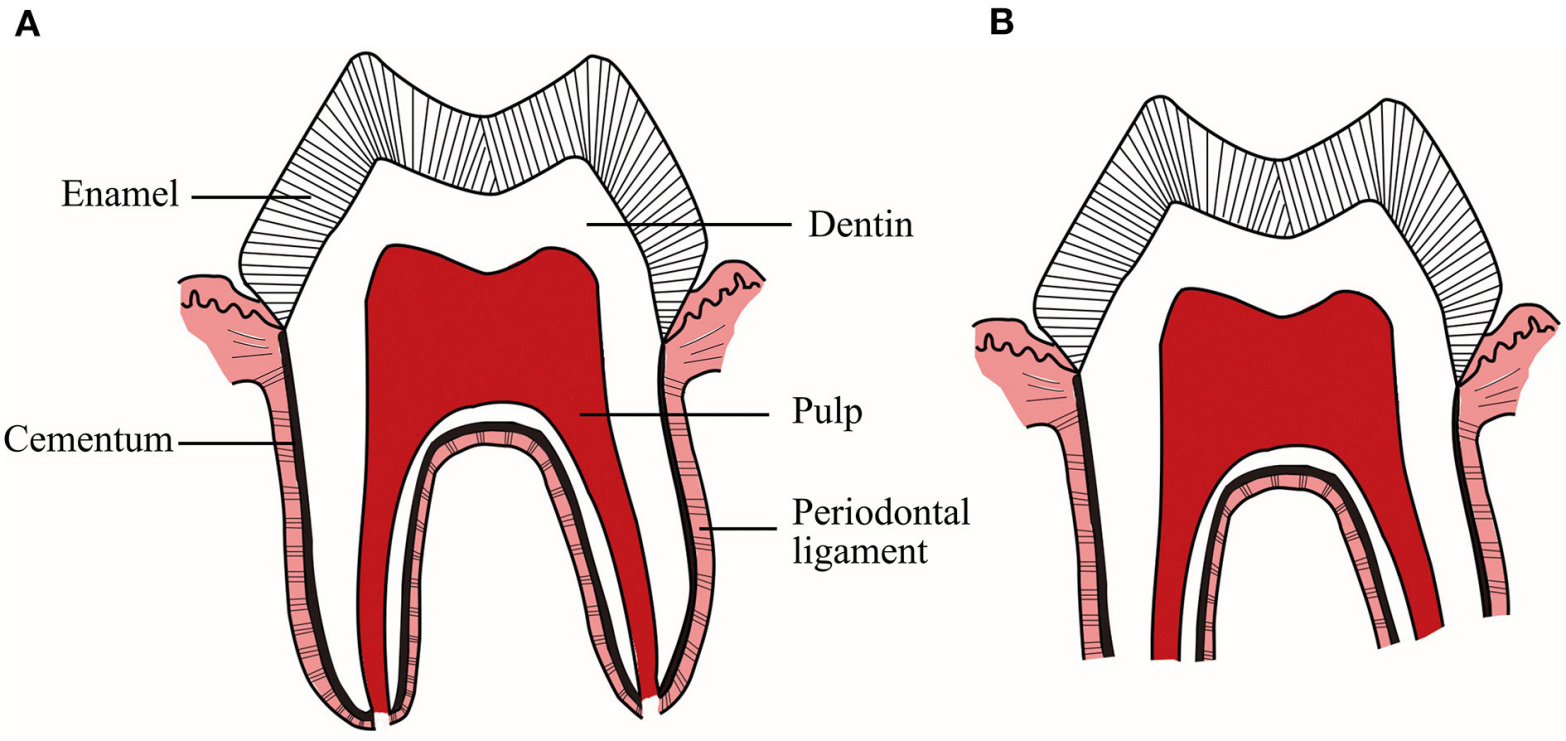
**(A)** Deciduous teeth with intact roots. **(B)** Deciduous teeth during physiological root resorption. This physiological process includes not only the resorption of hard tissues, such as dentin and cementum, but also the elimination of soft tissues, such as pulp and periodontal ligament.

Physiological root resorption of deciduous teeth, whose mechanisms are not yet clear, is a complex physiological process regulated by multiple cytokines and transcription factors through many different signaling pathways. As early as more than a decade ago, some scholars summarized and prospected the molecular and histological events of physiological root resorption (4). Since then, there are still few researches on the mechanisms related to physiological root resorption, but some new advances have been made. Accordingly, it is necessary to make a timely summary and review. Since this physiological process mainly includes the resorption of hard tissues, such as dentin and cementum, and the elimination of soft tissues, such as pulp and PDL (Figure 1), this article will review the research progress on the mechanisms of physiological root resorption from two aspects: hard tissues and soft tissues of deciduous teeth.

## Hard Tissues of Deciduous Teeth

### The Resorption of Hard Tissues in Deciduous Teeth

The resorption of hard tissues in deciduous teeth mainly involves osteoclast-mediated alveolar bone remodeling and odontoclast-mediated root resorption. In addition to the controversy over the presence and function of calcitonin receptors in odontoclasts, it is generally believed that odontoclasts and osteoclasts share similar biological properties and mechanisms of action (5, 6). Recent advances related to physiological resorption of the hard tissues in deciduous teeth mainly include the possible regulatory role of inflammatory microenvironment and mechanical stress in this process.

#### Inflammatory Microenvironment

**Figure 2:**
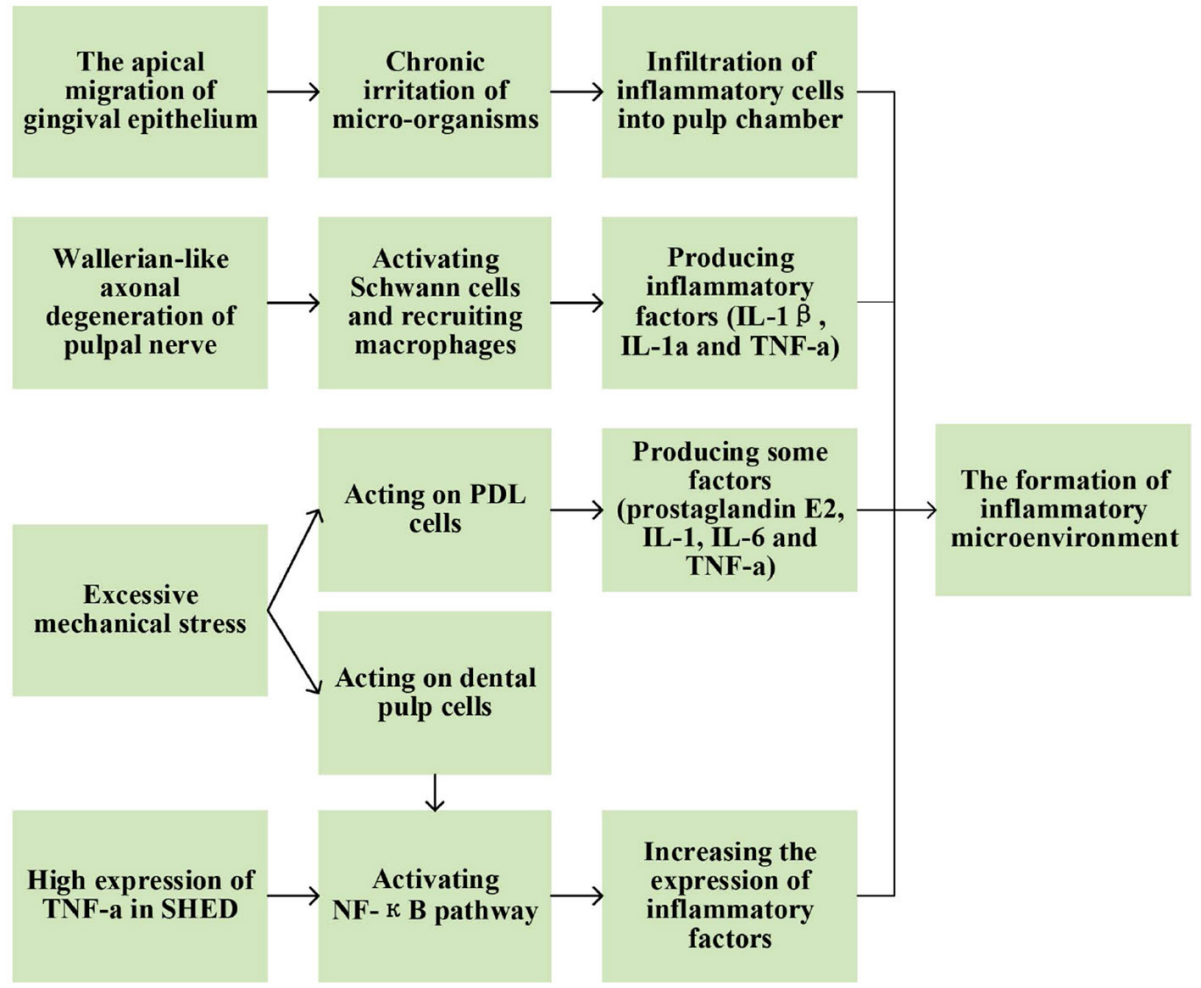
Possible factors contributing to the formation of inflammatory microenvironment during physiological root resorption.

Several factors may contribute to the formation of inflammatory microenvironment during physiological root resorption (Figure 2).

As physiological root resorption progressed, inflammatory cells, such as monocytes, macrophages and lymphocytes, gradually infiltrated into the pulp chamber (7, 8) with increasing density (9). This inflammatory microenvironment might arise from the apical migration of gingival epithelium during physiological root resorption. The presence of micro-organisms in the epithelial area might create a chronic irritation of dental pulp, leaving it in an inflammatory state (10). In addition, Wallerian-like axonal degeneration could be observed in pulpal nerve during this physiological process (11), the specific process and mechanisms of which will be described in the relevant section below. During Wallerian degeneration, activated Schwann cells and recruited macrophages also produced inflammatory cytokines, such as interleukin (IL)-1β, IL-1α and tumor necrosis factor-α (TNF-α) (12–14), which might also contribute to the formation of inflammatory microenvironment. However, it remains unclear how the inflammatory microenvironment affects physiological root resorption. In recent years, several studies have focused on some specific inflammatory factors to explore their expression and possible mechanisms in physiological root resorption.

##### TNF-α

TNF-α existed in stem cells from human exfoliated deciduous teeth (SHED) during physiological root resorption, and its expression in SHED was significantly higher than that in dental pulp stem cells from permanent teeth. In addition, TNF-α might regulate the expression of receptor-activator of nuclear-factor-κB ligand (RANKL) and osteoprotegerin (OPG) by activating nuclear factor-κB (NF-κB) pathway, which ultimately enhanced the ability of SHED to promote osteoclastogenesis (15), suggesting that TNF-α might be involved in the regulation of hard tissues resorption in deciduous teeth during physiological root resorption. The NF-κB signaling pathway is closely related to immune and inflammatory responses. Specifically, inflammatory factors, such as IL-1 and TNF-α, could activate NF-κB, which in turn acted as a key regulator of pro-inflammatory gene expression after activation, resulting in increased expression of these inflammatory factors (16, 17). This may also be relevant to the formation and role of inflammatory microenvironment in physiological root resorption.

##### TGF-β

In a study investigating whether dental pulp cells of deciduous teeth influenced osteoclastogenesis or odontoclastogenesis through the secretion of some key factors, the expression of transforming growth factor-β (TGF-β) was detected in the dental pulp cells of exfoliated deciduous teeth. Although there were no significant group differences, scholars pointed out that it could not rule out the possible importance of TGF-β in physiological root resorption (18). Shimazaki et al. investigated the possible role of TGF-β in periradicular tissues in regulating the differentiation of odontoclasts during physiological root resorption. The results showed that TGF-β1 and small amount of TGF-β3 were the main isoforms in the periradicular tissues of deciduous teeth. However, neither of them was the main factor affecting RANKL-mediated odontoclast differentiation, and this physiological process might be regulated by other cytokines (19). It follows that although TGF-β is closely associated with the differentiation of osteoclast or odontoclast (20, 21), it may not be the main regulatory factor affecting the progress of physiological root resorption.

##### ILs

In the inflammatory microenvironment of physiological root resorption, the expression of ILs, mainly including IL-1β and IL-6, was detected and tended to increase with the progress of root resorption (22, 23). Numerous studies have indicated that these ILs could play a crucial role in bone metabolism and root resorption in coordination with the receptor-activator of NF-κB (RANK)/RANKL/OPG system (24–26). This suggests that IL-1β and IL-6 may be involved in regulating the differentiation and maturation of osteoclast and odontoclast during physiological root resorption. However, the specific mechanisms of these ILs in physiological root resorption have not been reported.

#### Mechanical Stress

During masticatory movements and eruption of permanent teeth, deciduous teeth are subjected to various mechanical stresses. As the face grows and develops, the mechanical stresses exerted on the deciduous teeth gradually increase, possibly affecting the process of physiological root resorption. On the one hand, PDL cells can convert mechanical stimuli to biological signals and regulate the balance between osteogenesis and osteoclastogenesis. However, if subjected to excessive mechanical stress, the PDL cells would locally produce some factors, such as prostaglandin E2, IL-1, IL-6, and TNF-α, which might promote osteoclastogenesis and root resorption by upregulating the expression of RANKL in PDL cells (27–29). On the other hand, the mechanical stresses were also transmitted to the dental pulp, which had an impact on the intracellular signaling, proliferation, bone metabolism of dental pulp cells (30, 31). Therefore, both of these aspects may play a regulatory role in physiological root resorption.

##### PDL of Deciduous Teeth

Periodontal ligament stem cells (PDLSCs) in deciduous teeth promoted osteoclast differentiation through Runt-related transcription factor 2 (RUNX2), leading to increased physiological root resorption (32). RUNX2 is a key transcription factor in bone remodeling as well as root resorption. In children with Cleidocranial Dysplasia caused by RUNX2 mutation, retained deciduous teeth due to abnormal physiological root resorption and bone remodeling could often be observed (33, 34). This suggests that RUNX2 may be a crucial regulator in physiological root resorption. Considering the influence of chewing force, Chen et al. applied different degrees of mechanical stress to PDLSCs of deciduous teeth at different resorption stages to simulate the oral mechanical circumstances. The results showed that mechanical stress might activate the classic Wnt pathway by upregulating alpha 7 nicotinic acetylcholine receptor (a7 nAChR), a negative regulator of bone mass, resulting in a significant decrease in the expression of RUNX2, alkaline phosphatase (ALP) and OPG, and a significant increase in the expression of RANKL, which in turn induced osteoclast differentiation. Furthermore, researchers pointed out that chewing force might play a major role in the initiation of physiological root resorption (35, 36). In contrast to the results of this study, a large number of studies had actually shown the role of Wnt pathway in promoting osteoblast differentiation and inhibiting osteoclast differentiation (37–39). However, in an inflammatory environment, the activated Wnt pathway could inhibit osteoblast differentiation of PDLSCs (40). Therefore, it remains to be investigated whether the inflammatory microenvironment of physiological root resorption can also have the same effect on this pathway, causing PDLSCs of deciduous teeth to exhibit a role in inducing osteoclast differentiation.

In addition, the expression and specific role of RUNX2 at different resorption stages of deciduous teeth, the specific mechanism by which mechanical stress regulates the expression of a7 nAChR, and whether other cytokines and signaling pathways are involved in the regulation of physiological root resorption by PDL cells under mechanical stress can be further explored in the future.

##### Dental Pulp of Deciduous Teeth

During physiological root resorption, the dental pulp cells of deciduous teeth mediated monocyte-macrophage lineage to form osteoclasts or odontoclasts through the RANK/RANKL/OPG system. In this physiological process, the classic Wnt pathway might regulate the expression of RANKL and OPG, affecting the progress of physiological root resorption (18, 41). Under mechanical stress, the expression of secretory mammalian ly-6 urokinase-type plasminogen activator receptor-associated protein 1 (SLURP-1) in dental pulp stem cells of deciduous teeth was upregulated. SLURP-1 acted as an endogenous ligand to specifically activate a7 nAChR, enhancing the expression of NF-κB and significantly increasing the RANKL/OPG ratio. This sequence of events ultimately enhanced the ability of dental pulp stem cells in deciduous teeth to promote osteoclast differentiation, which might be associated with physiological root resorption and exfoliation of deciduous teeth (42).

It can be seen that PDL cells and dental pulp cells of deciduous teeth often involve the same factors or signaling pathways, such as a7 nAChR and Wnt pathway, when involved in the regulation of physiological root resorption. There may be a coordinated mechanism between the cells from PDL and dental pulp, which still needs temporal and spatial tracing of relevant factors to clarify the relationship between this two in regulating physiological root resorption. In addition, during inflammation, NF-κB and Wnt signaling pathway might also interact with each other, forming a complex regulatory network (43). Therefore, the study of physiological root resorption needs to consider the coordinated and integrated effects of various contributing factors, such as inflammatory microenvironment and mechanical stress, and different signaling pathways, such as NF-κB and Wnt signaling pathway.

### The Repair of Hard Tissues in Deciduous Teeth

The process of physiological root resorption is intermittent, which also includes the periods of remodeling and neoformation of dental structures in addition to the period of resorption (44). In the final stage of physiological root resorption, the resorbed surface of dentin or enamel in the pulp chamber could be partially or completely repaired by the deposition of cementum-like tissue (7, 44, 45). Localized proliferation of cementum was also seen during the resting period of resorption (8). In a study by Turkkahraman et al., cementum repair and reattachment of Sharpey's fibers were observed at the resorption lacunae of the root, which implied that the newly formed cementum might have reestablished the connection between the tooth and the PDL. During this physiological repair process, Wnt-responsive stem or progenitor cells derived from PDL might play a role, and Wnt/β-catenin signaling might be involved (46). In addition to Wnt/β-catenin signaling, some other signals, such as TGF-β signaling and FGF signaling, might also play an important regulatory role in cementum formation (47, 48). However, whether these signals can also be involved in regulating cementum repair in the context of physiological root resorption needs further investigation. In conclusion, physiological resorption of hard tissues is accompanied by physiological repair of hard tissues in deciduous teeth. More importantly, studying the mechanisms involved in this repair process may provide therapeutic ideas for the retention of deciduous teeth.

## Soft Tissues of Deciduous Teeth

### The Elimination of Soft Tissues in Deciduous Teeth

Rodrigues et al. proposed that apoptosis might be involved in the elimination of dental pulp during physiological root resorption (49). In order to better understand this event, they further explored the apoptotic pathway, suggesting that apoptosis of dental pulp cells in this physiological process was more likely to occur through the activation of caspase-3 by the mitochondrial pathway, an intrinsic pathway (50). The current understanding of the signaling system in apoptosis is still not comprehensive, and the relatively clear pathways of apoptosis mainly include intrinsic pathway mediated by mitochondria and extrinsic pathway mediated by death receptor (51). On this basis, Qian et al. further explored the possible pathways of apoptosis in dental pulp stem cells during physiological root resorption, and found that caspase-3, caspase-8 and caspase-9 were highly expressed in dental pulp stem cells of deciduous teeth compared to that of permanent teeth. Furthermore, the down-regulation of caspase-9 resulted in a significant decrease in apoptosis level, while down-regulation of caspase-8 resulted in no significant difference. This result also demonstrated the importance of caspase-9-mediated intrinsic pathway. However, whether the extrinsic pathway mediated by caspase-8 plays a role needs more research (52). In addition, when comparing the odontoblasts activity in the pulp of deciduous teeth with the pulp of permanent teeth, it was found that odontoblasts in deciduous teeth showed more apoptosis, leading to a reduction of odontoblastic layer, which also provides a new idea of research on the apoptotic mechanism of odontoblasts during physiological root resorption (53).

As mentioned above, the expression of TNF-α, IL-1β, and IL-6 was upregulated during physiological root resorption. TNF-α can induce apoptosis by activating caspase-8 through TNFR1 signaling pathway (54). IL-1β and IL-6 can also play a role in inducing apoptosis (55, 56). This suggests that the enhanced expression of these factors in physiological root resorption may promote the elimination of soft tissues in deciduous teeth by inducing apoptosis, but no relevant studies have been found so far.

### The Histological Changes of Soft Tissues in Deciduous Teeth

During physiological root resorption, corresponding changes in the nerve, blood vessels and other tissue structures were also observed. It had been noted that the blood vessels in the pulp maintained their normal structure at all stages of physiological root resorption (57), whereas most subsequent studies shown different results. At the end of physiological root resorption, increased vascularity, vascular dilatation and congestion, and increased blood flow in the dental pulp could be clearly observed, which might be related to the high metabolic demand of odontoclasts (44, 58, 59).

In addition to vascular changes, decreased innervation density in the pulp was also observed at the end of resorption (58), indicating that physiological root resorption might result in the denervation of pulp tissue. In the early stages of resorption, the peripheral nerve network of pulp remained well-developed. With the progress of physiological root resorption, a significant increase in neurofilament fragmentation was observed in the pulp tissue, accompanied by a gradual loss of myelinated axons, which might explain the declining sensory function of deciduous teeth in the pre-exfoliative stage. The mechanism of the decrease in pulpal innervation might mainly involve Wallerian-like axonal degeneration, an orderly process (11). In brief, it was the initial degradation of the axonal cytoskeleton, followed by the activation of myelinating Schwann cells that released myelin. When activated, on the one hand, Schwann cells could exhibit autophagic activity of phagocytosing myelin debris, and on the other hand, they could produce soluble factors that recruited immune cells, such as leukocytes, macrophages and blood-derived monocytes, to further remove myelin and axon debris, which was conducive to the recovery of injured nerves (60). In addition, Schwann cells played an important role in promoting peripheral nerve regeneration and functional recovery (61, 62), which was reflected in the early and middle stages of physiological root resorption (63). During these two stages, the expression of glial fibrillary acidic protein (GFAP) and growth-associated protein 43 (GAP-43) increased. The expression of GFAP was related to the proliferation of Schwann cells and the formation of Büngner bands, along which the damaged axons could grow and regenerate to reach the sites of innervation, whereas GAP-43 was generally expressed in the regenerated axons to direct the regeneration of injured nerves (11). In the advanced stages of physiological root resorption, nerve repair has not been reported. This might be due to the possibility that in the long-term state of denervation, Schwann cells were more likely to undergo apoptosis, making it difficult to support regeneration (64, 65). Therefore, it can be seen that the degeneration and loss of pulpal axons is accompanied by limited axonal regeneration and nerve repair during physiological root resorption. The specific mechanisms of nerve repair and how to regulate this repair process are directions that can be further investigated.

Although still controversial, it is generally believed that throughout the process of physiological root resorption, the histological changes of dental pulp tissue are not significant. Dental pulp in deciduous teeth can retain its potential for healing, repair and sensation until the advanced stages of root resorption (58, 66). In addition to providing further insight into the mechanisms of physiological root resorption, studying the histological changes of soft tissues in deciduous teeth during physiological root resorption can also understand the potential response of deciduous teeth to injury at different stages of resorption, which will facilitate the assessment of the prognosis of some clinical treatment measures, such as indirect pulp capping and pulpotomy, making a more sensible choice in the management of primary dentition.

## Conclusions

In conclusion, there are still many directions for further discussion regarding physiological root resorption, and relevant explanations have been given in each part above. All the studies mentioned above were carried out in the presence of permanent successors. In deciduous teeth without successors, physiological root resorption could also occur but started later and developed more slowly. At the onset of physiological root resorption in this type of deciduous teeth, a significant increase in T lymphocytes could be observed in the odontoblast layer of the apical pulp. In contrast, only a very small number of T lymphocytes were observed in the dental pulp and PDL of deciduous teeth with successors, suggesting that the immune system might play an important role in the initiation of physiological root resorption and that internal root resorption might play a more significant role in deciduous teeth without successors (67, 68). However, studies related to physiological root resorption in the absence of permanent successors are still scarce, which is also a major direction for future research and may provide ideas for retaining deciduous teeth without successors.

Due to the presence of inherited permanent teeth, many people tend to ignore the importance of deciduous teeth. However, abnormal resorption and exfoliation of deciduous teeth will also affect the normal development of permanent teeth. Therefore, the management of primary dentition is of great significance, and the study of physiological root resorption can provide a theoretical basis for better management of primary dentition in clinical practice.

## Author Contributions

MX, JL, and PW devised the review and the main conceptual ideas. MX reviewed the literature, drafted, and edited the manuscript. HQ critically revised the manuscript. All authors contributed to the article and approved the submitted version.

## Conflict of Interest

The authors declare that the research was conducted in the absence of any commercial or financial relationships that could be construed as a potential conflict of interest.

## Publisher's Note

All claims expressed in this article are solely those of the authors and do not necessarily represent those of their affiliated organizations, or those of the publisher, the editors and the reviewers. Any product that may be evaluated in this article, or claim that may be made by its manufacturer, is not guaranteed or endorsed by the publisher.
